# The Small Molecule PPARγ Agonist GL516 Induces Feeding-Stimulatory Effects in Hypothalamus Cells Hypo-E22 and Isolated Hypothalami

**DOI:** 10.3390/molecules27154882

**Published:** 2022-07-30

**Authors:** Annalisa Chiavaroli, Viviana di Giacomo, Barbara De Filippis, Amelia Cataldi, Claudio Ferrante, Letizia Giampietro

**Affiliations:** Department of Pharmacy, “G. d’Annunzio” University, Via dei Vestini 31, 66100 Chieti, Italy; annalisa.chiavaroli@unich.it (A.C.); viviana.digiacomo@unich.it (V.d.G.); barbara.defilippis@unich.it (B.D.F.); amelia.cataldi@unich.it (A.C.); claudio.ferrante@unich.it (C.F.)

**Keywords:** PPARγ agonists, fibrates, neuroprotection, hypothalamus, small molecules, neuropeptides, neurotransmitters

## Abstract

PPARγ agonists are implicated in the regulation of diabetes and metabolic syndrome and have therapeutic potential in brain disorders. PPARγ modulates appetite through its central effects, especially on the hypothalamic arcuate nucleus (ARC). Previous studies demonstrated that the small molecule GL516 is a PPARγ agonist able to reduce oxidative stress and apoptosis with a potential neuroprotective role. Herein, we investigated the effects of GL516, in vitro and ex vivo, on the levels of hypothalamic dopamine (DA) and serotonin (5-HT). The gene expressions of neuropeptide Y, CART, AgRP, and POMC, which play master roles in the neuroendocrine regulation of feeding behavior and energy balance, were also evaluated. HypoE22 cells were treated with H_2_O_2_ (300 μM) for 2 h e 30’ and with different concentrations of GL516 (1 nM-100 µM). The cell viability was evaluated after 24 and 48 h of culturing using the MTT test. DA and 5-HT levels in the HypoE22 cell supernatants were analyzed through HPLC; an ex vivo study on isolated hypothalamic specimens challenged with scalar concentrations of GL516 (1–100 µM) and with pioglitazone (10 µM) was carried out. The gene expressions of CART, NPY, AgRP, and POMC were also determined by a quantitative real-time PCR. The results obtained showed that GL516 was able to reduce DA and 5-HT turnover; moreover, it was effective in stimulating NPY and AgRP gene expressions with a concomitant reduction in CART and POMC gene expressions. These results highlight the capability of GL516 to modulate neuropeptide pathways deeply involved in appetite control suggesting an orexigenic effect. These findings emphasize the potential use of GL516 as a promising candidate for therapeutical applications in neurodegenerative diseases associated with the reduction in food intake and stimulation of catabolic pathways.

## 1. Introduction

Peroxisome proliferator-activated receptors (PPARs) are ligand-activated transcription factors that include three isoforms encoded by PPARα, PPARγ, and PPARβ/δ. PPARs play a crucial role in the regulation of lipid metabolism and glucose homeostasis [1]. PPARs are expressed in almost all mammalian tissues and organs and the expression patterns of the three isoforms are different with some intersections. In particular, high expression levels of PPARs are detected in active fatty acid metabolism tissues [2]. In recent years, the growing importance of PPARs involved in the central nervous system (CNS) functions has been demonstrated. It is possible to find all three PPARs expressed in the amygdala and the hypothalamus [3], whereas PPARγ is also expressed in neurons, astrocytes, and other glial cells [4].

The implications of PPARs in the pathologies of the CNS mainly refer to the regulation of the inflammatory processes in microglia and macrophages and the inhibition of the production of cytokines and other inflammatory mediators [5]. Lately, the involvement of PPAR ligands in neurodegenerative disorders, such as Alzheimer’s disease (AD), multiple sclerosis, amyotrophic lateral sclerosis (ALS), and Parkinson’s disease (PD) was well established [6,7,8]. In particular, accumulating evidence suggests that, in addition to diabetes and metabolic syndrome, PPARγ agonists have significant therapeutic potential in brain disorders. Experimental studies and clinical observations suggest that PPARγ ligands may be successfully used to treat neurological diseases, such as neurodegenerative diseases, as well as traumatic injuries, stroke, and demyelinating diseases [9,10,11].

Neurodegenerative disorders are often correlated with appetite disturbances. For example, the loss of appetite can possibly occur as a consequence of defective central and peripheral appetite regulation in patients with ALS [12]. In addition, there is evidence for hypothalamic degeneration in patients with this pathology, as well as in non-symptomatic individuals who carry genetic risk factors for ALS [13]. Although the loss of appetite is a significant factor in the weight loss of patients with ALS, it is not associated with other clinical features typical of the disease, such as impairment of respiratory functions. Therefore, loss of appetite can be related to the involvement of central mechanisms of control of this function [14]. For these reasons, in patients with neurodegenerative disorders who exhibit loss of appetite, early intervention for weight maintenance that promotes appetite to slow weight loss could be of clinical significance.

Previous studies demonstrated that PPARγ modulates appetite in both rodents and humans through its central effects, exerted especially on the hypothalamic arcuate nucleus (ARC) [15,16,17,18].

In particular, Matias et al. [19] demonstrated that central injection of the PPARγ agonist rosiglitazone increases food consumption in domesticated chicks but does not affect water intake. This effect may be associated with changes in transcriptional regulation of both orexigenic (NPY) and anorexigenic (POMC) factors within the hypothalamus. 

The hypothalamus has long been known to be involved in regulating feeding behavior through the interplay between peripheral afferents, namely adipokines, gut-derived peptides, neurotransmitters, and neuropeptides [20,21,22]. Specifically, neuropeptide Y (NPY) and agouti-related peptide (AgRP) co-expressing neurons in the hypothalamic arcuate nucleus stimulate feeding, whilst proopiomelanocortin (POMC) and cocaine- and amphetamine-regulated transcript (CART) peptide co-expressing neurons induce anorexigenic behavior [23]. Additionally, dopamine (DA), norepinephrine (NE), and serotonin (5-hydroxytryptamine, 5-HT) play modulatory roles in feeding control in the hypothalamus [24]. 

In the search for new PPAR agonists, we synthesized some small molecules, fibrate derivatives, which were found to act as PPARγ or PAN agonists [25,26]. Indeed, the typical PPARγ agonists are thiazolidinediones, and fibrates are usually considered a class of antihyperlipidemic agents that act through PPARα activation [27]. The fibrate scaffold, with different chemical modifications, also provides a wide spectrum of biological activities [28]; compounds containing a fibrate scaffold have shown different properties [29], also independent from PPARα activation, such as anti-inflammatory, analgesic [30], antioxidant [31,32], and antiplatelet activities [33].

**Figure 1:**
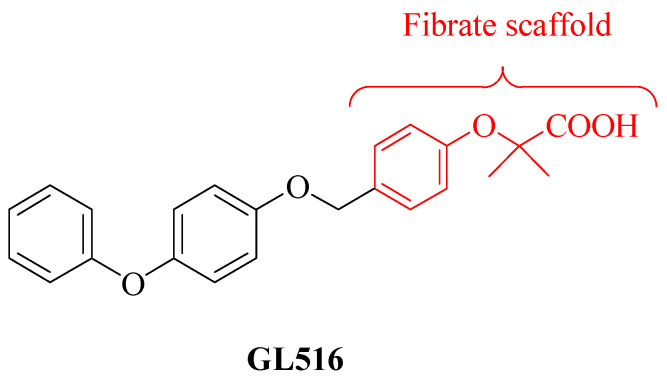
Chemical structure of the PPARγ agonist GL516.

With regard to these activities, our group reported the ability of the fibrate analog PPARγ agonist GL516 (Figure 1) to reduce oxidative stress and apoptosis in a rat astrocyte cell line, suggesting a potential role of this small molecule in the prevention of neurodegenerative disorders [34].

Based on these assumptions, and in order to better understand the role of GL516 in promoting appetite, especially in patients with neurodegenerative disorders, the aim of the present study was to evaluate the effects of this compound at the hypothalamic level. For this purpose, hypothalamic HypoE22 cells and isolated hypothalamic specimens were employed for evaluating GL516 biocompatibility and its effects on the levels of hypothalamic neurotransmitters, such as dopamine and serotonin, respectively. Moreover, in hypothalamic tissue, the gene expressions of neuropeptide Y, CART, AgRP, and POMC, all of which play master roles in the neuroendocrine regulation of feeding behavior and energy balance, were also investigated [23]. 

## 2. Results and Discussion

**Figure 2:**
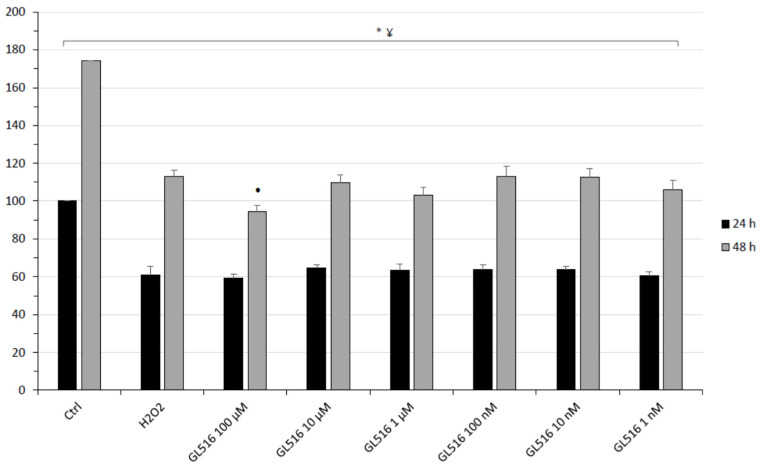
MTT viability test of the rat hypothalamic cell line HypoE22 exposed to different concentrations of GL516 (1 nM–100 μM) for 24 (black bars) and 48 (grey bars) hours. * *p* > 0.05 vs. Ctrl 24 h; ¥ *p* > 0.05 vs. Ctrl 48 h; • GL516 vs. H_2_O_2_ 48 h.

In the present study, the neuroprotective and neuromodulatory properties of the compound GL516 were analyzed in vitro and ex vivo. Specifically, hypothalamic HypoE22 cells and isolated tissues were selected, as previously described [35,36]. Formerly, GL516 was tested in HypoE22 cells, in the presence of hydrogen peroxide, 300 µM, chosen as the pro-oxidant stimulus. In the range 1 nM–100 µM, GL516 was not effective in contrasting the decrease of cell viability induced by hydrogen peroxide (Figure 2). 

Being the MTT assay is not very sensitive, apoptosis detection was evaluated in the same experimental conditions, but no changes were found (data not shown). This could seem to contrast with already reported antioxidant properties [34]. However, this apparent discrepancy can be explained by the different cell line model; in the previous one, the experimental model was a cell line of rat astrocytes, confirming the key role of these types of glial cells in neuron physiology [37]. 

PPAR agonists, besides neuroprotection, have also been suggested to modulate feeding behavior and hypothalamic neuromodulators [15].

**Figure 3:**
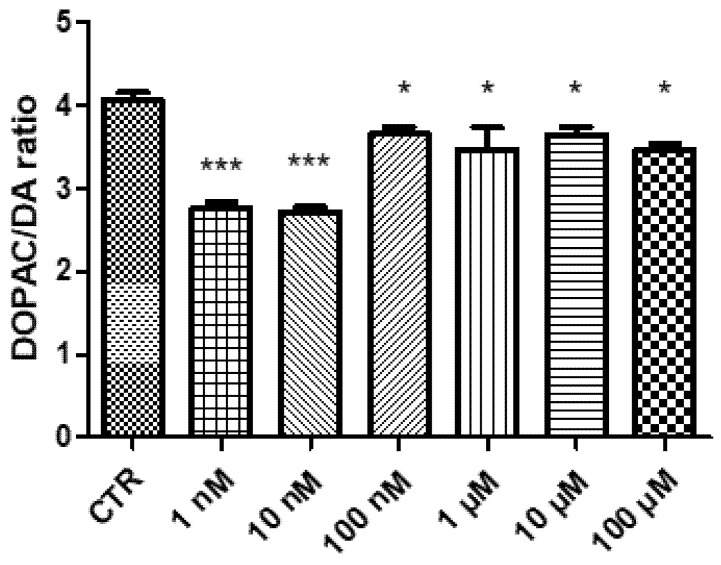
Inhibitory effect induced by GL516 on dopamine turnover (DOPAC/DA ratio) in the isolated hypothalamus exposed to GL516 for 4 h. ANOVA, *p* < 0.0001; * *p* < 0.05, *** *p* < 0.001, vs. control (CTR).

**Figure 4:**
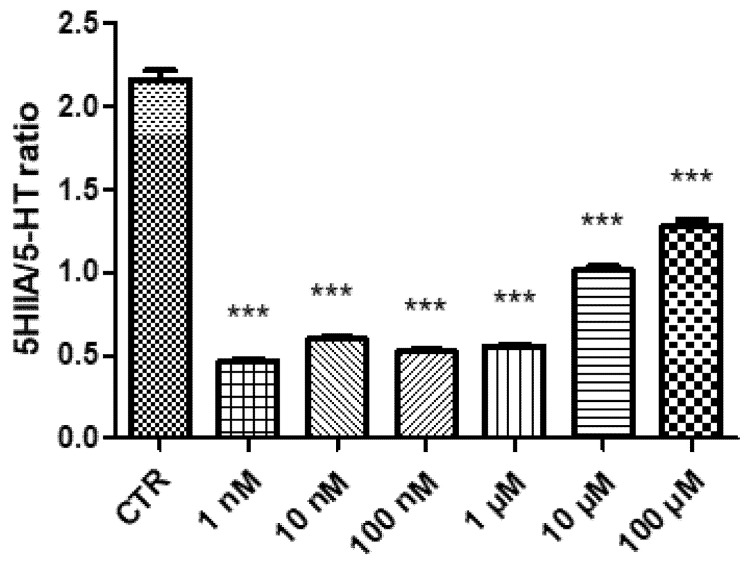
Inhibitory effect induced by GL516 on serotonin turnover (5HIIA/5-HT ratio) in the isolated hypothalamus exposed to GL516 for 4 h. ANOVA, *p* < 0.0001; *** *p* < 0.001, vs. control (CTR).

In the present study, the effects of GL516 on hypothalamic aminergic neurotransmitters, namely dopamine and serotonin, were investigated as well. Specifically, GL516 was able to reduce DA and 5-HT turnover (Figure 3 and Figure 4), expressed as the DOPAC/DA and 5HIIA/5-HT ratio, respectively. The increase in DA and 5-HT levels was particularly evident at the lowest tested concentration, with a progressive reduction with the increase of the dosage; thus, scaling back the importance of these neurotransmitters as hypothalamic mediators for GL516 effects. 

However, the results were more relevant when GL516 was assayed on isolated hypothalamic specimens comparing its activity with pioglitazone (PGZ). Pioglitazone, belonging to the class of thiazolidinediones (TZDs), is a PPARγ agonist used for diabetic patients as an insulin-sensitizing agent. PGZ reduces the mortality causes and the occurrence of non-fatal myocardial infarction and stroke in patients with type 2 diabetes mellitus (DM2) and a high risk of macrovascular events [38]. PGZ has been defined as a “polypharmacy in a pill” because it is able to have a neuroprotective effect through the modulation of inflammation, oxidative stress, microglial defects, cerebral glucose consumption, and mitochondrial functions. Interestingly, the neuroprotective effects of PGZ are also strongly correlated with its ability to lower insulin levels in non-diabetic patients [39]. For these reasons, PGZ was used as the reference compound to explore the activity of our PPARγ agonist GL516.

**Figure 5:**
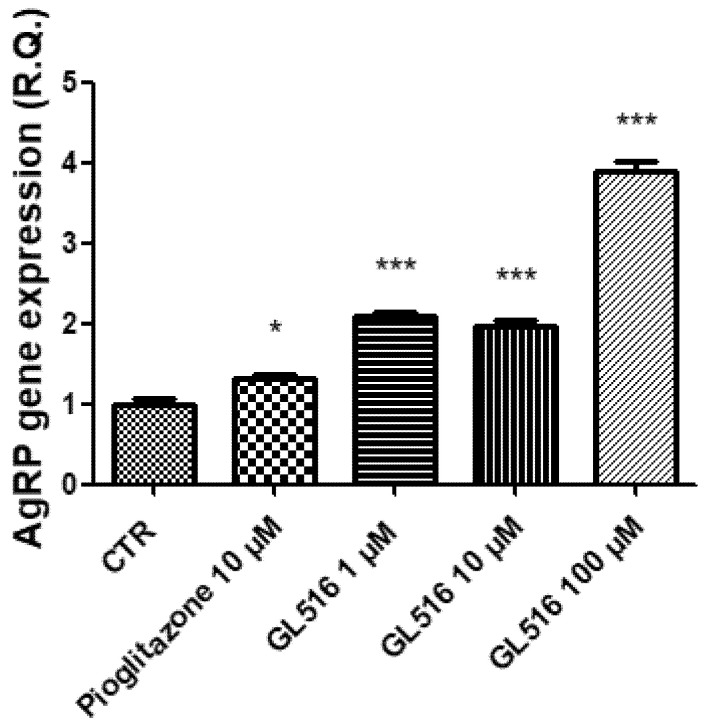
Stimulatory effects induced by GL516 on the AgRP gene expression in the isolated hypothalamus exposed to GL516 for 4 h. ANOVA, *p* < 0.0001; * *p* < 0.05, *** *p* < 0.001, vs. control (CTR).

**Figure 6:**
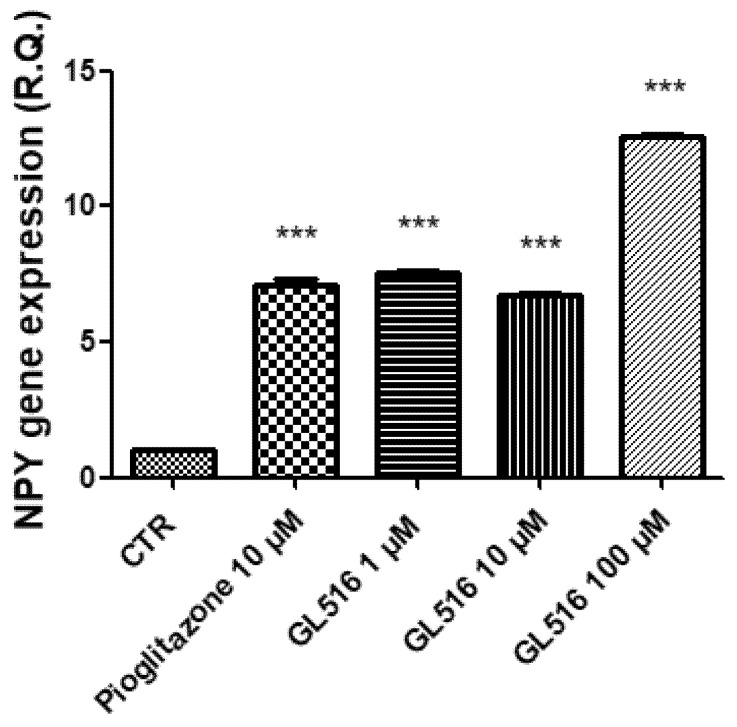
Stimulatory effects induced by GL516 on the NPY gene expression in the isolated hypothalamus exposed to GL516 for 4 h. ANOVA, *p* <0.0001; *** *p* < 0.001, vs. control (CTR).

**Figure 7:**
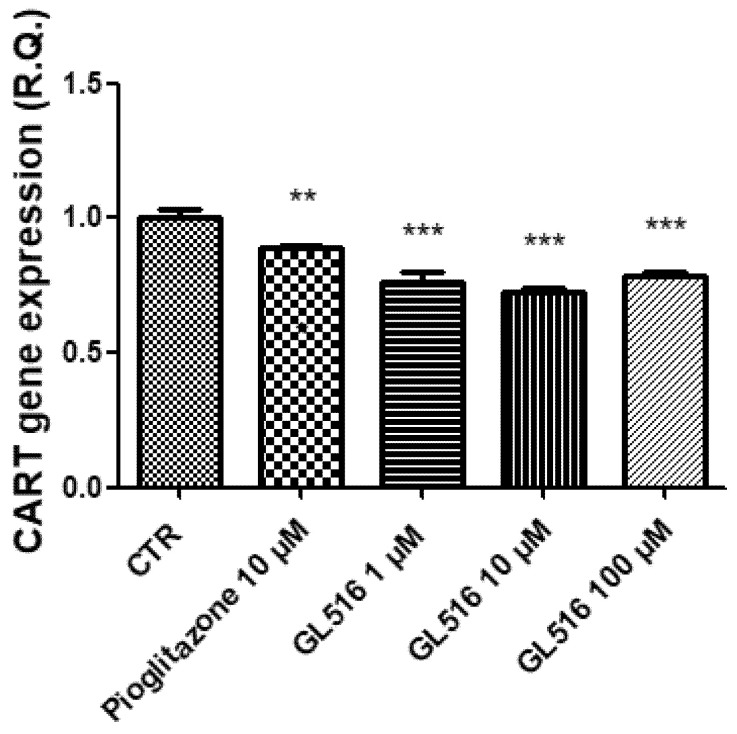
Inhibitory effects induced by GL516 on the CART gene expression in the isolated hypothalamus exposed to GL516 for 4 h. ANOVA, *p* < 0.0001; ** *p* < 0.01, *** *p* < 0.001, vs. control (CTR).

**Figure 8:**
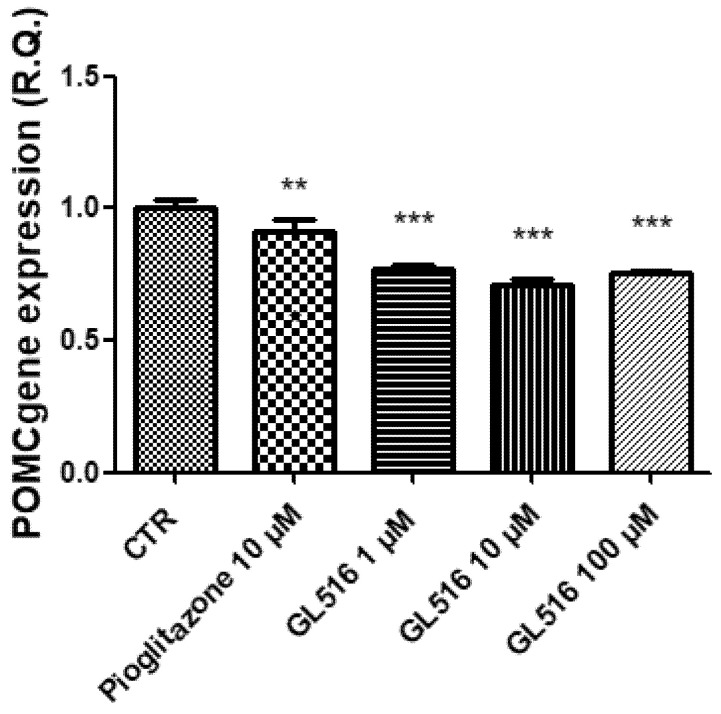
Inhibitory effects induced by GL516 on the POMC gene expression in the isolated hypothalamus exposed to GL516 for 4 h. ANOVA, *p* <0.0001; ** *p* <0.01, *** *p* <0.001, vs. control (CTR).

In the ex vivo model, the gene expressions of NPY, AgRP, CART, and POMC were evaluated by treating the tissue with a scalar concentration of GL516 (1–100 μM) and with PGZ (10 μM). In agreement with previous studies conducted on PPAR agonists, GL516 was effective at stimulating NPY and AgRP gene expressions (Figure 5 and Figure 6), with a concomitant reduction in CART and POMC gene expressions (Figure 7 and Figure 8). 

In particular, as shown in Figure 5, GL516 was able to significantly stimulate the AgRP gene expression more than pioglitazone. Interestingly, GL516 induces an increase in the AgRP gene expression already at 1 μM as compared to PGZ at 10 μM. This effect is more evident at the highest concentration.

Moreover, treatments at 1 and 10 μM of GL516 are able to cause a significant increase in the NPY gene expression comparable with PGZ at 10 μM (Figure 6). Moreover, in this case, the increase in gene expression is more evident at the highest concentration. 

Regarding CART and POMC, GL516 showed a slight reduction of the gene expression referred to as PGZ even at a lower concentration (1 μM) and in a non-dose-dependent manner (Figure 7 and Figure 8).

These results highlight the ability of GL516 to modulate neuropeptide pathways deeply involved in appetite control better than PGZ. Additionally, the present findings suggest a putative orexigenic effect induced by the compound. This would indicate a potential use of GL516 as a promising candidate, or lead compound, for the development of new treatments against neurodegenerative diseases, which are often paralleled by a concomitant reduction in food intake and stimulation of catabolic pathways.

In this context, future in vivo studies need to be conducted to validate the present findings and the past results obtained by in vitro studies, pointing out the antioxidant and neuromodulatory properties of GL516 [34].

As a concluding remark, and highlighting the importance of such a compound as an innovative protective treatment in neurodegenerative diseases—an in silico prediction was conducted on the bioinformatics platform SwissTargetPrediction. The analysis was focused on the capability of GL516 to bind to multiple target proteins, thus further supporting the multidirectional mechanism of this compound, which could explain, albeit partially, the observed in vitro effects (Supplementary Material). Indeed, besides being predicted to interact with PPARs, the primary target for this compound, GL516, appears, according to the in silico prediction, to also interact with TRPM8, which is known to be involved in energy balance control in the hypothalamus; thus, further strengthening the hypothesis of a feeding-stimulatory effect exerted by GL516.

## 3. Materials and Methods

### 3.1. Hypothalamic HypoE22 cells: Evaluation of Neuromodulatory Effects

Hypothalamic HypoE22 cells were purchased from Cedarlane Cellution Biosystems (Burlington, ON, Canada) and cultured in DMEM (Euroclone) supplemented with 10% (*v*/*v*) heat-inactivated fetal bovine serum and 1.2% (*v*/*v*) penicillin G/streptomycin in a 75 cm^2^ tissue culture flask (no = 5 individual culture flasks for each condition). The culture conditions and the viability 3-(4,5-dimethylthiazol-2-yl)-2,5-diphenyltetrazolium bromide (MTT) test were performed as previously described [36]. 

The cells were seeded on 96-well plates at a density of 5 × 103 cells/well. When indicated, the cells were treated with H_2_O_2_ (300 μM) for 2 h e 30′ and with different concentrations of GL516 (1 nM–100 µM). The cell viability was evaluated after 24 and 48 h of culturing using the MTT (3–[4,5–dimethylthiazol–2–yl– T2,5–diphenyl tetrazolium bromide) growth assay (Sigma-Aldrich, St. Louis, MO, USA), based on the capability of viable cells to reduce MTT to a colored formazan product. The MTT (10 µL; 0.5 mg/mL) was added to each well and the plates were incubated at 37 °C for 3 h. Subsequently, this solution was replaced by 100 µL of DMSO and the reduced MTT was quantized at a wavelength of 570 nm on an ELISA reader (Bio-Rad, Hercules, CA, USA). 

Effects of the GL516 (1 nM–100 µM) on cell viability were evaluated in comparison to the untreated control (Ctrl) group.

### 3.2. Quantitative Determination of Dopamine and Serotonin 

DA and 5-HT levels in the HypoE22 cell supernatants were analyzed through an HPLC apparatus consisting of a Jasco (Tokyo, Japan) PU-2080 chromatographic pump and an ESA (Chelmsford, MA, USA) Coulochem III coulometric detector, equipped with a microdialysis cell (ESA-5014b) porous graphite working electrode and solid state palladium reference electrode. The detailed description of the chromatographic analysis is fully described in our previous study [35]. 

### 3.3. Ex Vivo Studies

Adult C57/BL6 male mice (3 months old, weight 20–25 g) were housed in Plexiglas cages (2–4 animals per cage; 55 cm × 33 cm × 19 cm) and maintained under standard laboratory conditions (21 ± 2 °C; 55 ± 5% humidity) on a 14/10 h light/dark cycle, with ad libitum access to water and food. Housing conditions and experimental procedures were strictly in agreement with the European Community ethical regulations (EU Directive no. 26/2014) on the care of animals for scientific research. In agreement with the recognized principles of “Replacement, Refinement and Reduction in Animals in Research”, isolated hypothalami were obtained as residual material from vehicle-treated mice randomized in our previous experiments, approved by the local ethical committee (‘G. d’Annunzio University, Chieti, Italy) and the Italian Health Ministry (project no. 885/2018-PR).

Isolated hypothalamic specimens were maintained in a humidified incubator with 5% CO_2_ at 37 °C for 4 h (incubation period), in an RPMI buffer. During the incubation period, the tissues were challenged with scalar concentrations of GL516 (1–100 µM) and with pioglitazone (10 µM)

### 3.4. RNA Extraction, Reverse Transcription, and Real-Time Reverse Transcription Polymerase Chain Reaction (RT-PCR)

Total RNA was extracted from hypothalamic specimens using TRI reagent (Sigma-Aldrich, St. Louis, MO, USA), according to the manufacturer’s protocol, and reverse transcribed using High Capacity cDNA Reverse Transcription Kit (Thermo Fisher Scientific, Waltman, MA, USA). The gene expressions of CART, NPY, AgRP, and POMC were determined by quantitative real-time PCR using TaqMan probe-based chemistry, as previously described [35,40]. PCR primers and TaqMan probes were obtained from Life Technologies (Assays-on-Demand Gene Expression Products), Mm00475829_g1 for AgRP gene (hypothalamus), Mm03048253_m1 for NPY gene (hypothalamus), Mm00489086_m1 for CART gene (hypothalamus), and Mm00435874_m1 for POMC gene (hypothalamus). β-actin was used as the housekeeping gene. The data elaborations were conducted with the Sequence Detection System (SDS) software version 2.3 (Thermo Fisher Scientific). Relative quantification of gene expression was performed by the comparative 2^−∆∆Ct^ method (Livak and Schmittgen, 2001).

### 3.5. Statistical Analysis

Statistical analyses were performed using GraphPad Prism version 5.01 software (San Diego, CA, USA). Means ± S.E.M. were determined for each experimental group and analyzed by a one-way analysis of variance (ANOVA), followed by Newman–Keuls comparison multiple tests. Statistical significance was set at *p* < 0.05.

## 4. Conclusions

To conclude, the present study explored the potential role of GL516, a novel synthetic PPAR agonist as a neuromodulatory agent in the hypothalamus. Specifically, GL516 was effective at reducing biogenic amine turnover and anorexigenic peptide gene expression, with a concomitant increase in the gene expressions of hypothalamic orexigenic neuropeptides.

Together with previous findings pointing to protective effects in the brain [34], the present results point to future applications of GL516 and congeners as novel treatments for counteracting neurodegenerative diseases, often characterized by an imbalance in feeding stimulatory pathways.

## Data Availability

The data presented in this study are available upon request from the corresponding author.

## Supplementary Materials

The following supporting information can be downloaded at: https://www.mdpi.com/article/10.3390/molecules27154882/s1, In silico prediction.

## Sample Availability

Samples of the compound GL516 are available upon request from the corresponding author.
