# Supplementary material for: The Small Molecule PPARγ Agonist GL516 Induces Feeding-Stimulatory Effects in Hypothalamus Cells Hypo-E22 and Isolated Hypothalami
Source: Molecules. 2022 Jul 30;27(15):4882. doi: 10.3390/molecules27154882 (PMC9369729; doi:10.3390/molecules27154882)
Supplement: Supplementary file 1 [file molecules-27-04882-s001.zip › molecules-1819355-supplementary.pdf]

SwissTargetPrediction

Target	Common name	Uniprot ID	ChEMBL ID	Target Class	Probability*	Known actives (3D/2D)
T1R1/T1R3_UNCURATED	TAS1R3 TAS1R1	Q7RTX0 Q7RTX1	CHEMBL3832641	Family C G protein-coupled receptor	0,153290397	0 / 1
Peroxisome proliferator-activated receptor gamma	PPARG	P37231	CHEMBL235	Nuclear receptor	0,113285953	979 / 49
Peroxisome proliferator-activated receptor alpha	PPARA	Q07869	CHEMBL239	Nuclear receptor	0,113285953	590 / 48
Aldose reductase	AKR1B1	P15121	CHEMBL1900	Enzyme	0,113285953	370 / 0
Carnitine O-palmitoyltransferase 1, liver isoform	CPT1A	P50416	CHEMBL1293194	Enzyme	0,113285953	198 / 0
Carnitine O-palmitoyltransferase 1, muscle isoform	CPT1B	Q92523	CHEMBL2216739	Group translocator	0,113285953	136 / 0
Angiotensin-converting enzyme	ACE	P12821	CHEMBL1808	Protease	0,113285953	394 / 0
5-lipoxygenase activating protein	ALOX5AP	P20292	CHEMBL4550	Other cytosolic protein	0,113285953	242 / 0
Neprilysin	MME	P08473	CHEMBL1944	Protease	0,113285953	340 / 0
Carboxypeptidase A1	CPA1	P15085	CHEMBL2088	Protease	0,113285953	19 / 0
Angiotensin-converting enzyme 2	ACE2	Q9BYF1	CHEMBL3736	Protease	0,113285953	40 / 0
Kelch-like ECH-associated protein 1	KEAP1	Q14145	CHEMBL2069156	Unclassified protein	0,113285953	12 / 0
Matrix metalloproteinase 2	MMP2	P08253	CHEMBL333	Protease	0,113285953	292 / 0
Carboxypeptidase B	CPB1	P15086	CHEMBL2552	Protease	0,113285953	17 / 0
Liver glycogen phosphorylase	PYGL	P06737	CHEMBL2568	Enzyme	0,113285953	135 / 0
Signal transducer and activator of transcription 3 (	STAT3	P40763	CHEMBL4026	Transcription factor	0,113285953	18 / 0
Phospholipase A2 group 1B	PLA2G1B	P04054	CHEMBL4426	Enzyme	0,113285953	59 / 0
Lysosomal protective protein	CTSA	P10619	CHEMBL6115	Protease	0,113285953	370 / 0
Neurotensin receptor 1	NTSR1	P30989	CHEMBL4123	Family A G protein-coupled receptor	0,113285953	28 / 0
Integrin alpha-4/beta-1	ITGB1 ITGA4	P05556 P13612	CHEMBL1907599	Membrane receptor	0,113285953	646 / 0
Phosphodiesterase 4B	PDE4B	Q07343	CHEMBL275	Phosphodiesterase	0,113285953	141 / 0
Phosphodiesterase 4D	PDE4D	Q08499	CHEMBL288	Phosphodiesterase	0,113285953	107 / 0
Matrix metalloproteinase 9	MMP9	P14780	CHEMBL321	Protease	0,113285953	131 / 0
Casein kinase II alpha	CSNK2A1	P68400	CHEMBL3629	Kinase	0,113285953	149 / 0
Leukocyte elastase	ELANE	P08246	CHEMBL248	Protease	0,113285953	40 / 2
p53-binding protein Mdm-2	MDM2	Q00987	CHEMBL5023	Other nuclear protein	0,113285953	181 / 0
Dihydroorotate dehydrogenase	DHODH	Q02127	CHEMBL1966	Oxidoreductase	0,113285953	281 / 0
Endothelin receptor ET-A (by homology)	EDNRA	P25101	CHEMBL252	Family A G protein-coupled receptor	0,113285953	468 / 0
Glycogen synthase kinase-3 beta	GSK3B	P49841	CHEMBL262	Kinase	0,113285953	88 / 0
Free fatty acid receptor 1	FFAR1	O14842	CHEMBL4422	Family A G protein-coupled receptor	0,113285953	223 / 0
Chymase	CMA1	P23946	CHEMBL4068	Protease	0,113285953	124 / 0
Cathepsin G	CTSG	P08311	CHEMBL4071	Protease	0,113285953	15 / 0
Hexokinase type IV	GCK	P35557	CHEMBL3820	Enzyme	0,113285953	43 / 0
Sodium channel protein type X alpha subunit (by h	SCN10A	Q9Y5Y9	CHEMBL5451	Voltage-gated ion channel	0,113285953	28 / 0
Carnitine palmitoyltransferase 2	CPT2	P23786	CHEMBL3238	Enzyme	0,113285953	62 / 0
Caspase-1	CASP1	P29466	CHEMBL4801	Protease	0,113285953	221 / 0
Endothelin receptor ET-B	EDNRB	P24530	CHEMBL1785	Family A G protein-coupled receptor	0,113285953	171 / 0
Integrin alpha-V/beta-3	ITGAV ITGB3	P06756 P05106	CHEMBL1907598	Membrane receptor	0,113285953	318 / 0

Protein farnesyltransferase	FNTA FNTB	P49354 P49356	CHEMBL2094108	Enzyme	0,113285953	110 / 0
Integrin alpha-V/beta-5	ITGB5 ITGAV	P18084 P06756	CHEMBL2096675	Membrane receptor	0,113285953	30 / 0
Integrin alpha-V/beta-1	ITGAV ITGB1	P06756 P05556	CHEMBL2111407	Membrane receptor	0,113285953	7 / 0
Integrin alpha-V/beta-6	ITGAV ITGB6	P06756 P18564	CHEMBL2111416	Membrane receptor	0,113285953	8 / 0
Dual specificity protein phosphatase 3	DUSP3	P51452	CHEMBL2635	Phosphatase	0,113285953	34 / 0
Matrix metalloproteinase 13	MMP13	P45452	CHEMBL280	Protease	0,113285953	234 / 0
Matrix metalloproteinase 3	MMP3	P08254	CHEMBL283	Protease	0,113285953	157 / 0
Sn1-specific diacylglycerol lipase alpha	DAGLA	Q9Y4D2	CHEMBL5545	Enzyme	0,113285953	18 / 0
Integrin alpha-4/beta-7	ITGB7 ITGA4	P26010 P13612	CHEMBL2095184	Membrane receptor	0,113285953	209 / 0
Aldehyde reductase	AKR1A1	P14550	CHEMBL2246	Enzyme	0,113285953	18 / 0
ADAMTS5	ADAMTS5	Q9UNA0	CHEMBL2285	Protease	0,113285953	96 / 0
Autotaxin	ENPP2	Q13822	CHEMBL3691	Enzyme	0,113285953	37 / 0
Matrix metalloproteinase 14	MMP14	P50281	CHEMBL3869	Protease	0,113285953	59 / 0
Matrix metalloproteinase 12	MMP12	P39900	CHEMBL4393	Protease	0,113285953	102 / 0
Phosphodiesterase 10A	PDE10A	Q9Y233	CHEMBL4409	Phosphodiesterase	0,113285953	44 / 0
Matrix metalloproteinase 8	MMP8	P22894	CHEMBL4588	Protease	0,113285953	130 / 0
Neurokinin 2 receptor	TACR2	P21452	CHEMBL2327	Family A G protein-coupled receptor	0,113285953	5 / 0
Integrin alpha2/beta1	ITGB1 ITGA2	P05556 P17301	CHEMBL3137268	Unclassified protein	0,113285953	30 / 0
Heat shock protein HSP 90-alpha	HSP90AA1	P07900	CHEMBL3880	Other cytosolic protein	0,113285953	21 / 0
Neurokinin 3 receptor	TACR3	P29371	CHEMBL4429	Family A G protein-coupled receptor	0,113285953	3 / 0
Bone morphogenetic protein 1	BMP1	P13497	CHEMBL3898	Protease	0,113285953	18 / 0
Oxoeicosanoid receptor 1	OXER1	Q8TDS5	CHEMBL1628461	Family A G protein-coupled receptor	0,113285953	4 / 0
c-Jun N-terminal kinase 1	MAPK8	P45983	CHEMBL2276	Kinase	0,113285953	38 / 0
MAP kinase signal-integrating kinase 2	MKNK2	Q9HBH9	CHEMBL4204	Kinase	0,113285953	26 / 0
6-phosphofructo-2-kinase/fructose-2,6-bisphosphat	PFKFB3	Q16875	CHEMBL2331053	Enzyme	0,113285953	18 / 0
Matrix metalloproteinase 1	MMP1	P03956	CHEMBL332	Protease	0,113285953	89 / 0
Protein-tyrosine phosphatase 1B	PTPN1	P18031	CHEMBL335	Phosphatase	0,113285953	212 / 0
Receptor-type tyrosine-protein phosphatase F (LAR	PTPRF	P10586	CHEMBL3521	Membrane receptor	0,113285953	34 / 0
T-cell protein-tyrosine phosphatase	PTPN2	P17706	CHEMBL3807	Phosphatase	0,113285953	56 / 0
PI3-kinase p110-alpha subunit	PIK3CA	P42336	CHEMBL4005	Enzyme	0,113285953	51 / 0
Steroid 5-alpha-reductase 2	SRD5A2	P31213	CHEMBL1856	Oxidoreductase	0,113285953	66 / 0
Receptor-type tyrosine-protein phosphatase beta	PTPRB	P23467	CHEMBL2706	Phosphatase	0,113285953	3 / 0
Integrin alpha-4	ITGA4	P13612	CHEMBL278	Membrane receptor	0,113285953	106 / 0
Glycogen synthase kinase-3 alpha	GSK3A	P49840	CHEMBL2850	Kinase	0,113285953	20 / 0
Protein-tyrosine phosphatase 1C	PTPN6	P29350	CHEMBL3166	Phosphatase	0,113285953	11 / 0
Protein-tyrosine phosphatase G1	PTPN12	Q05209	CHEMBL3236	Phosphatase	0,113285953	2 / 0
Leukocyte common antigen	PTPRC	P08575	CHEMBL3243	Enzyme	0,113285953	15 / 0
Serine/threonine-protein kinase Aurora-A	AURKA	O14965	CHEMBL4722	Kinase	0,113285953	71 / 0
Prostanoid EP4 receptor	PTGER4	P35408	CHEMBL1836	Family A G protein-coupled receptor	0,113285953	235 / 0
Peptidyl-prolyl cis-trans isomerase NIMA-interacting	PIN1	Q13526	CHEMBL2288	Enzyme	0,113285953	57 / 0

Chymotrypsin C	CTRC	Q99895	CHEMBL2386	Protease	0,113285953	3 / 0
Metabotropic glutamate receptor 2 (by homology)	GRM2	Q14416	CHEMBL5137	Family C G protein-coupled receptor	0,113285953	19 / 0
Transient receptor potential cation channel subfam	TRPM8	Q7Z2W7	CHEMBL1075319	Voltage-gated ion channel	0,113285953	91 / 0
Integrin alpha-5/beta-1	ITGB1 ITGA5	P05556 P08648	CHEMBL2095226	Membrane receptor	0,113285953	39 / 0
Bromodomain-containing protein 4	BRD4	O60885	CHEMBL1163125	Reader	0,113285953	15 / 0
Replication protein A 70 kDa DNA-binding subunit	RPA1	P27694	CHEMBL1764940	Unclassified protein	0,113285953	20 / 0
Epidermal growth factor receptor erbB1	EGFR	P00533	CHEMBL203	Kinase	0,113285953	61 / 0
Fatty acid binding protein adipocyte	FABP4	P15090	CHEMBL2083	Fatty acid binding protein family	0,113285953	81 / 0
Cyclin-dependent kinase 9	CDK9	P50750	CHEMBL3116	Kinase	0,113285953	14 / 0
Fatty acid binding protein muscle	FABP3	P05413	CHEMBL3344	Fatty acid binding protein family	0,113285953	33 / 0
Fatty acid binding protein epidermal	FABP5	Q01469	CHEMBL3674	Fatty acid binding protein family	0,113285953	15 / 0
Bradykinin B1 receptor	BDKRB1	P46663	CHEMBL4308	Family A G protein-coupled receptor	0,113285953	4 / 0
3-phosphoinositide dependent protein kinase-1	PDPK1	O15530	CHEMBL2534	Kinase	0,113285953	5 / 0
Gonadotropin-releasing hormone receptor	GNRHR	P30968	CHEMBL1855	Family A G protein-coupled receptor	0,113285953	13 / 0
Epoxide hydratase	EPHX2	P34913	CHEMBL2409	Protease	0,113285953	87 / 0
Nerve growth factor receptor Trk-A	NTRK1	P04629	CHEMBL2815	Kinase	0,113285953	17 / 0
Phosphoglycerate kinase 1	PGK1	P00558	CHEMBL2886	Enzyme	0,113285953	2 / 0
Vitronectin receptor alpha	ITGAV	P06756	CHEMBL3660	Membrane receptor	0,113285953	30 / 0
L-lactate dehydrogenase A chain	LDHA	P00338	CHEMBL4835	Enzyme	0,113285953	51 / 0
L-lactate dehydrogenase B chain	LDHB	P07195	CHEMBL4940	Enzyme	0,113285953	18 / 0
Egl nine homolog 3	EGLN3	Q9H6Z9	CHEMBL5705	Enzyme	0,113285953	1 / 0
Lysine-specific demethylase 2A	KDM2A	Q9Y2K7	CHEMBL1938210	Eraser	0,113285953	24 / 0
